# Clinical decision support system for quality of life among the elderly: an approach using artificial neural network

**DOI:** 10.1186/s12911-022-02044-9

**Published:** 2022-11-12

**Authors:** Maryam Ahmadi, Raoof Nopour

**Affiliations:** Department of Health Information Management, School of Health Management and Information Sciences, Iran University of Medical Sciences, Tehran, Iran

**Keywords:** Elderly, Quality of life, Clinical decision support system, Artificial neural network, Machine learning

## Abstract

**Background:**

Due to advancements in medicine and the elderly population’s growth with various disabilities, attention to QoL among this age group is crucial. Early prediction of the QoL among the elderly by multiple care providers leads to decreased physical and mental disorders and increased social and environmental participation among them by considering all factors affecting it. So far, it is not designed the prediction system for QoL in this regard. Therefore, this study aimed to develop the CDSS based on ANN as an ML technique by considering the physical, psychiatric, and social factors.

**Methods:**

In this developmental and applied study, we investigated the 980 cases associated with pleasant and unpleasant elderlies QoL cases. We used the BLR and simple correlation coefficient methods to attain the essential factors affecting the QoL among the elderly. Then three BP configurations, including CF-BP, FF-BP, and E-BP, were compared to get the best model for predicting the QoL.

**Results:**

Based on the BLR, the 13 factors were considered the best factors affecting the elderly’s QoL at *P* < 0.05. Comparing all ANN configurations showed that the CF-BP with the 13-16-1 structure with sensitivity = 0.95, specificity  =  0.97, accuracy = 0.96, F-Score = 0.96, PPV = 0.95, and NPV = 0.97 gained the best performance for QoL among the elderly.

**Conclusion:**

The results of this study showed that the designed CDSS based on the CFBP could be considered an efficient tool for increasing the QoL among the elderly.

## Background

The new advancement in the field of medicine and the improvement of people’s health caused the number of older adults to increase worldwide [1]. Aging is the natural stage in the human lifespan, occurring in the latest stages of life and leading to physiological, psychological, and social changes in a person [2]. According to studies, the growth of the elderly population is such that it will reach more than two billion by 2050 [3]. There is no consensus about the age at which the elderly started in the countries, and it differs concerning their hygienic and socioeconomic conditions [4]. According to the World Health Organization (WHO) announcement, the elderly age is considered 65 years old and above [5]. This age group is more prone to chronic diseases such as hypertension, hypercholesterolemia, and diabetes. Older adults over 70 are associated with more severe disease burdens such as myocardial infarction, stroke, cancer, and chronic obstructive pulmonary disease [6]. Also, by increasing the elderly population and chronic diseases, mental disorders, including schizophrenia, psychiatric dementia, and bipolar disorders, and social limitations, such as increasing dependence on others in performing personal tasks, would be increased [7, 8]. So, attention to the elderly’s QoL is essential in their latest years of life [9, 10]. The QoL is a sophisticated concept related to the physical, mental, environmental, and social factors among the elderly [11, 12]. Improving the QoL among the elderly is crucial [13]. However, behaviors for enhancing health and QoL among the elderly are essential subjects that have not been considered much in today's societies [14]. Evaluating the QoL among the elderly in clinical settings leads to closer cooperation between physicians, patients, and other care providers. This assessment increases patients’ awareness of their diseases and health status, familiarizes them with the advantages and disadvantages of various treatments, and emphasizes the patient’s role in selecting treatment approaches [15]. Prediction models have played an essential role in evaluating and improving elderly health by increasing the speed and accuracy of predicting their physical, mental, and social health [16].

On the other hand, there are many raw data in the field of health that, despite knowledge discovery methods, their role in increasing organizational insight has been ignored [17]. Predictive models based on machine learning methods have a significant role in identifying physical and psychological disorders, analyzing the factors affecting diseases, increasing the diagnostic accuracy of care providers, and reducing the need for invasive interventions for the early diagnosis of physicians [18, 19]. On the other hand, little research has been conducted concerning social disorders in people. There is still information in this field that lack of use has reduced evidence-based methods and incorrect decision-making in the field of timely prevention of these disorders [20, 21]. Predictive models using machine learning techniques make it possible to accurately assess the quality of life of the elderly, allowing more people to live their lives with greater ability to reduce falls [22]. ANNs as an ML method has significant roles in predicting various conditions with high capabilities, such as medical domains [23]. Different researchers applied the ANNs as a favorable method for prediction. Jahani et al. used the ANNs to predict the effects of human activities on vegetation diversity. They concluded that the ANNs had a strong ability for the prediction process in this respect [24]. The ANNs also gained high prediction performance in predicting the mortality risk among COVID-19 patients [25]. One study concluded that the ANNs is the best technique for building the prediction model for tree failure under windstorm than other ML algorithms [26]. Shahmoradi et al. used ANNs to forecast neurofeedback response in patients with Attention Deficit Hyperactivity Disorder (ADHD). They found that the ANNs could be utilized as a supportive tool in decision-making by different healthcare providers [27]. In one work, the ANNs also was used in a prediction system for detecting COVID-19 disease [28]. The CDSSs based on ANNs were used as a prediction system to easily detect hypericin content by pharmacognosists, managers, and producers [29]. The BP type of the ANNs is extensively used to solve many classification and prediction problems, especially considering the large amount of data in the medical domain [30]. CDSSs using ML techniques are practically and effectively used to predict various medical situations using the data, facilitating and making more straightforward clinical judgments and increasing clinical outcomes [31, 32]. Most previous studies in the applications of ML techniques in QoL investigated physical dysfunctions, psychiatric disorders, and social factors less analyzed in this respect [33–35]. Sue et al. investigated the elderly QoL using ML algorithms through depression as a behavioral factor [36]. Lee et al. constructed the prediction model using ML models for QoL among the elderly with chronic diseases [37]. Using ML techniques, Prati investigated the correlation between elderly QoL and psychological factors [38]. The factors influencing the elderly QoL, including demographic and physical and psychological characteristics, were investigated by Kwon [39]. As introduced by past studies, a few focused on social aspects for predicting the QoL among the elderly and considering the social and more varied factors could have a potential role in better prediction [40]. In this study, we apply social factors and others to increase our prediction model capability and generalizability. Therefore, we aim to design the CDSS based on the ANN as the ML technique for predicting the QoL among the elderly by comprehensively analyzing physical, psychiatric, and social factors. Also, the CDSS suggests the best solution for improving the QoL among the elderly based on the system results and assessing influencing factors.

## Methods

### Study framework

**Fig. 1:**
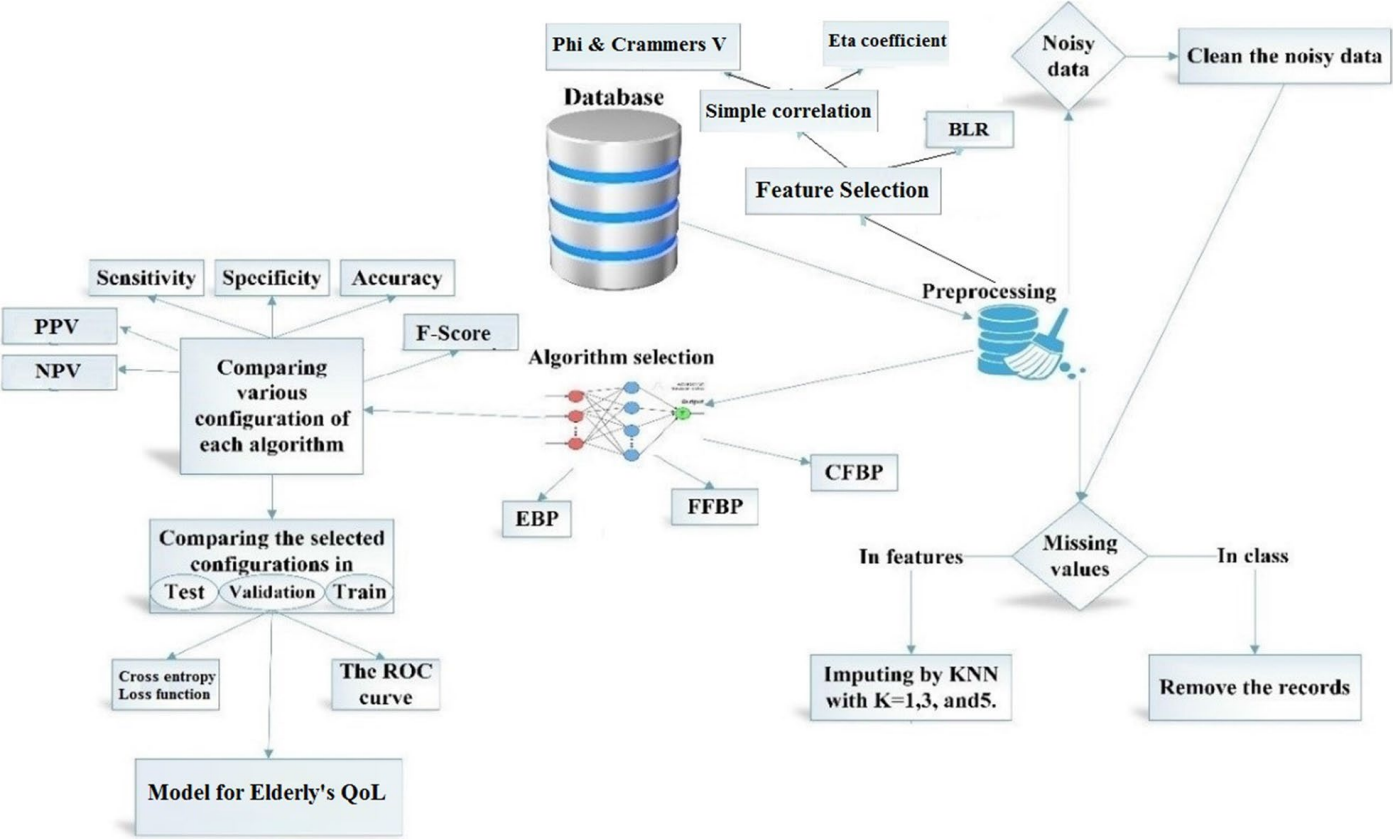
The roadmap of the study

This study is descriptive-applied and developmental type. It included four steps: dataset description, preprocessing of the dataset, adjusting the artificial neural network structure, and developing the CDSS for predicting the elderly’s QoL based on the best configuration. The methodology of this study is shown in Fig. 1.

In the dataset description step, we reported the duration and the centers in which data was gathered. We introduced the features in the dataset and described the scales and tools used for measuring some eligible features in the dataset. The frequency of each variable’s value was reported. Also, we analyzed our dataset in terms of existing missing and noisy values and duplicated records. In the preprocessing step, we replaced missing and noisy values in the dataset using the KNN method for independent variables and removed the records that did not have class labels. To get the most relevant factors for predicting the elderly’s QoL, we used the Phi and Crammers V and Eta correlation coefficient for simple correlation and BLR for correlation with output class in the presence of other independent input variables. Then, we selected three ANN algorithms and adjusted the parameters of the algorithms for the learning process in the next step. To obtain the best configuration in each algorithm, we compared the performance of algorithms using the sensitivity, specificity, accuracy, F-Score, PPV, and NPV. After obtaining the best configuration in each selected algorithm, we applied the ROC and binary cross entropy loss function in all training, testing, and validation modes to investigate the best structure in overfitting. Finally, we obtained the best model for predicting the QoL among the elderly.

### Dataset description

This study aims to predict QoL among the elderly using the ANN as an intelligent method. The study population was the elderly from three elderly centers in Ahvaz city, where their information was stored in one database from January 2019 to January 2021. The features in the database were categorized into four main classes: demographic data, disabilities and comorbidities, social factors, and various mental health indexes.

### Predictor features

#### Demographic factors

Age (more than 65 years old), gender, marital status, educational level, and place of residence.

#### Disabilities and comorbidities

Heart diseases and arterial blood pressure, musculoskeletal diseases, diabetes mellitus, gastrointestinal diseases, respiratory diseases, nervous diseases, cancer, mental disorders, visual dysfunction, hearing dysfunction, skin diseases, body pain, and other diseases and disabilities.

#### Independent living index

It refers to the ability of the elderly to respond to some activities in daily life. These abilities include basic and cognitive skills. Essential skills are eating, bathing/washing, dressing, grooming, toileting, mobility, and climbing. Cognitive skills include social and domestic activities. A five-point Likert scale determines the difficulty of doing daily activities for the elderly from one to five, where a score of one is equivalent to no problem, and five is considered very difficult. The sum of these scores shows the degree of dependence of the elderly in performing daily activities.

#### Perceived health status

This variable is measured by asking the question of people: How do you rate your health? Answers are presented in the form of a five-point Likert scale summarized into three groups. Options include very bad/bad = 0, average = 1, and good and excellent = 2.

#### Positive mood index

This factor includes mental status such as worries, aggressiveness, stress, anxiety, and tension, and they represent negative mental health states in people characterized by a three-point Likert scale including not at all, little, and very much.

#### Negative mood index

This factor includes states such as relaxation and enjoyment, and they express the desired psychological state of people determined by a three-point Likert scale, including none, low and high, measured with a five-point Likert scale with the options of never, once/twice a year, once/twice a month, once/twice a week, and every day.

#### Formal social cohesion

It is one of the social factors that include public meetings, meeting with community leaders, organizational meetings, and social meetings with neighbors.

#### Informal social cohesion

It refers to meeting friends at home, neighbors, and religious meetings. Social cohesion includes seven expressed social participations, measured with a five-point Likert scale with the options of never, once/twice a year, once/twice a month, once/twice a week, and every day.

#### Physical and mental abilities

Physical performance, vivacity, general health, and the ability to play an emotional role.

### Outcome variable

#### Quality of life among the elderly

It can be obtained from the five-point Likert scale and answers the question, how do you evaluate the quality of your life? The response to the question is categorized in five-point Likert scales, including “very bad,” “bad,” “moderate,” “good,” and “very good,” which were converted into two groups as “unfavorable” and “favorable” by gerontologists and stored in the database to reduce the groups for QoL assessment among the elderly.

### Dataset preparation

In this study, we first removed records that had missing values greater than 5% of missing data in their attribute values. For the features with missing values, we used the imputation method using the (K-nearest neighborhood) KNN algorithm with specific amounts of K = 1, 3, and 5 to replace the missing values. In the noisy data and the records with less than 5% missing values, we first removed them and replaced them using the KNN algorithm. In the second step, to increase the algorithm's performance, remove the irrelevant features and prevent overfitting of the algorithms, we used the feature selection process to gain the most factors affecting the QoL among the elderly. In this study, we used the Phi & Crammers V and Eta coefficients to get the variables affecting the QoL among the elderly as simple correlation coefficients. Also, BLR with the Enter method was applied to determine the highly correlated factors for the elderly's QoL in the presence of other independent factors. The *P* < 0.05 was considered a statistically meaningful level in this regard.

### Adjusting the artificial neural network structure

ANNs are mathematical models and abstract constructions that mimic the human brain [41]. They included the units named neurons for processing the data and calculation process. The ANN typically consists of input, hidden or computational, and output layers. The input layer receives the data from the environment and transfers it to the hidden layers consisting of many calculation neurons. Finally, the results of the calculations are transferred to the output layer to introduce to the users [42]. The transfer functions convert the weighted variables to increase the ANN performance by predicting the output variable [43]. A BP of ANN was used for the learning process and adjusting the weights and biases of the neurons. In the BP type of ANN learning, the difference between the ANNs output (QoL among the elderly) and actual output. In this type of ANN, the weight of nodes (W) and input variables (X) will be set to W_i_ and W_j_ for the i-th and jth of the input variable and neuron. The target of the Jth node on the Kth hidden layer (net_j^k) can be computed by the following Equation:

1 $${\text{net }}_{{\text{j}}}^{{\text{k}}} = { }\mathop \sum \limits_{{{\text{i}} = 0}}^{{\text{n}}} {\text{W}}_{{{\text{ij}}}} {\text{X}}_{{{\text{ij}}}}$$

To get the ANN’s target for elderly QoL, we used the Tansig function to transfer the ANN’s target to a normalized value. This function can be run in MATLAB with high speed, considering a few differences from other functions in performance. Therefore, Eq. 2 shows the ANN output for predicting the QoL among the elderly.

2 $${\text{Tansig }}\left( {{\text{net}}_{{\text{j}}}^{{\text{k}}} } \right)$$

We used the gradient descent function to calculate the best weight of neurons during training the ANN (Eq. 3). (W_ji_) and γ represent constant in Equation. The total squared error determines the differences between the ANN’s output and the actual result. When training in ANN finishes, the best weight will be assigned to the nodes by training functions, and the gradient will be minimized [24, 27].

3 $${\text{W}}_{{{\text{ji}}}}^{{\text{n}}} = {\text{ W}}_{{{\text{ji}}}}^{{{\text{n}} - 1}} + \left( { - {\upgamma }\frac{{\partial {\text{E}}^{{\text{t}}} }}{{\partial {\text{W}}_{{{\text{ji}}}}^{{\text{t}}} }}} \right)$$

In this study, after gaining the most important factors affecting the elderly’s QoL, we used the three configurations of CF-BP, FF-BP, and E-BP for training the ANN and building the predictive model for the elderly’s QoL. The 70%, 20%, and 10% of data were used for training, testing, and validation. We used the Tansig activation function for building the models in all configurations in the ANN’s input, hidden, and output layers. We used the Levenberg Marquardt (ML) method to train the ANN due to its high convergence speed, reduced training time, and optimal performance, especially in a large dataset [44]. The training time and the number of all ANN training iterations were set to unlimited and 1000, respectively. It was not a problem due to the high speed of the LM algorithm.

### Developing the CDSS for quality of life among the elderly

In this study, we first compared various modes in each ANN configuration by increasing the number of neurons in hidden layers to get the best ANN prediction structure. In this regard, we used sensitivity (Eq. 4), specificity (Eq. 5), accuracy (Eq. 6), F-Score (Eq. 7), PPV (Eq. 8), and NPV (Eq. 9). Next, we used the binary cross entropy loss function and the Area under the ROC curve (AUC) to compare the best mode in each configuration to investigate the performance criteria in each train, test, and validation mode in terms of overfitting the algorithms. Then, we obtained the best model for predicting the QoL among the elderly by comparing the best structure in each ANN mode in the train, test, and validation modes. At last, the CDSS for predicting the elderly’s QoL was designed in MATLAB V 2013-a.

4 $${\text{Sensitivity}} = \frac{{{\text{TP}}}}{{{\text{TP}} + {\text{FN}}}}$$

5 $${\text{Specificity}} = { }\frac{{{\text{TN}}}}{{{\text{TN}} + {\text{FP}}}}$$

6 $${\text{Accuracy}} = \frac{{{\text{TP}} + {\text{TN}}}}{{{\text{TP}} + {\text{FN}} + {\text{FP}} + {\text{TN}}}}$$

7 $${\text{F}} - {\text{Score}} = 2{*}\frac{{\text{Sensitivity*Specificity}}}{{\left( {{\text{Sensitivity}} + {\text{Specificity}}} \right)}}$$

8 $${\text{PPV}} = \frac{{{\text{TP}}}}{{{\text{TP}} + {\text{FP}}}}$$

9 $${\text{NPV}} = \frac{{{\text{TN}}}}{{{\text{TN}} + {\text{FN}}}}$$

### External validation cohort

In the last step of the study, we used the external validation process to investigate our predictive system’s generalizability. In this respect, we gathered 147 cases of QoL and non-QoL among the elderly. Of these, 65 cases and 82 cases were related to the quality of life of the elderly, respectively, and were related to two elderly centers named Panhkala-Jonubi and Mehravaran-Shamal in Sari province. We tested our prediction system with these samples and reported the performance of our model generalization using a confusion matrix. Then we compared the performance of the cohort's prediction system and the best CF-BP performance in this study using the ROC curve.

## 3. Results

### Preprocessing of the dataset

After removing the cases with more than 5% missing values in their attributes, 5 cases pertained to QoL, and non-QoL cases were removed from the study. The loss of data associated with the 67 cases, with less than 5%, was imputed using the KNN algorithm. So 975 cases remained in the study and were used for analysis. Among 975 cases, 346 and 629 cases belonged to the QoL and non-QoL of the elderly, respectively.

### Simple correlation analysis

The results of determining the correlation between each factor affecting the QoL among the elderly and QoL using the Crammers V and phi coefficient are shown in Table 1.

**Table 1:** Factors affecting the QoL with simple correlation

Variable	Values (%N) or Mean (SD)	Values (encodes)	Phi & V Crammers correlation/ Eta	*P*-value
Age	75.1 (5.88)	–	0.61	
Gender	Male (46.5%) Female (53.5%)	Male (0) Female (1)	0.17	0.25
Marital status	With wife (13%) Without wife (87%)	With wife (0) Without wife (1)	0.12	0.31
Educational level	No-literacy/Elementary (68%) Less than diploma (23%) Diploma/Academic (9%)	No-literacy/Elementary (1) Less than diploma (2) Diploma/Academic (3)	0.06	0.35
Place of residence	Urban (63%) Rural (37%)	Urban (0) Rural (1)	0.12	0.4
Heart diseases and arterial blood pressure	Yes (33%) No (67%)	Yes (1) No (0)	0.3	0.01
Musculoskeletal diseases	Yes (13%) No (87%)	Yes (1) No (0)	0.4	0.02
Gastrointestinal diseases	Yes (6%) No (94%)	Yes (1) No (0)	0.23	0.1
Respiratory diseases (asthma)	Yes (14%) No (86%)	Yes (1) No (0)	0.38	0.03
Nervous diseases	Yes (15%) No (85%)	Yes (1) No (0)	0.21	0.15
Cancer	Yes (6%) No (94%)	Yes (1) No (0)	0.33	0.04
Mental dysfunctions	Yes (39%) No (61%)	Yes (1) No (0)	0.3	0.02
Visual disability	Yes (23%) No (77%)	Yes (1) No (0)	0.39	0.01
Hearing disability	Yes (41%) No (59%)	Yes (1) No (0)	0.34	0.01
Skin diseases	Yes (12%) No (88%)	Yes (1) No (0)	0.18	0.16
Diabetes mellitus	Yes (40%) No (60%)	Yes (1) No (0)	0.15	0.02
Other diseases	Yes (24%) No (76%)	Yes (1) No (0)	0.08	0.21
Independent Living Index	Score 1 (18%) Score 2 (33%) Score 3 (32%) Score 4 (12%) Score 5 (5%)	Score 1 (1) Score 2 (2) Score 3 (3) Score 4 (4) Score 5 (5)	0.35	0.01
Physical performance	Pleasant (57%) Unpleasant (43%)	Pleasant (1) Unpleasant (0)	0.18	0.03
Body pain	Yes (46%) No (54%)	Yes (1) No (0)	0.12	0.03
Perceived health status	Very bad/Bad (6%) Moderate (58%) Good (36%)	Very bad/Bad (1) Moderate (2) Good (3)	0.1	0.04
Ability to emotional role	Yes (34.8%) No (65.2%)	Yes (1) No (0)	0.13	0.03
Positive mood index	None (39%) Low (45%) High (16%)	None (0) Low (1) High (2)	0.43	0.001
Negative mood index	None (13%) Low (29%) High (57.4%)	None (0) Low (1) High (2)	0.37	0.001
Formal social cohesion	Never (13%) One/two per year (33%) One/two per month (27%) One/two per week (20%) Every day (7%)	Never (0) One/two per year (1) One/two per month (2) One/two per week (3) Every day (4)	0.25	0.001
Informal social cohesion	Never (5%) One/two per year (15%) One/two per month (48%) One/two per week (28%) Every day (3%)	Never (0) One/two per year (1) One/two per month (2) One/two per week (3) Every day (4)	0.21	0.001
General health	Yes (73%) No (27%)	Yes (1) No (0)	0.13	0.04
Vitality	Yes (60%) No (40%)	Yes (1) No (0)	0.1	0.04

Table 1 shows the factors affecting the QoL among the elderly. This includes the frequency of each affecting factor reported by percent with codes defined in the database for each value of factors. The Mean (SD) of the age of the elderly was 75.1 ± 5.88 years. Also, 46.5% and 53.5% of the sample were associated with the men and women elderly, respectively. In this table, the simple correlation between each input factor and the elderly’s QoL was measured using the phi and Crammers V correlation and the Eta coefficient at a statistically significant level. By considering *P* < 0.05 and ŋ > 0.4 as the significant level for input variables statistically, the variables of age (ŋ = 0.61), heart diseases and arterial blood pressure (*P* = 0.01), musculoskeletal diseases (*P* = 0.02), respiratory diseases (*P* = 0.03), cancer (*P* = 0.04), mental dysfunction (*P* = 0.02), visual disability (*P* = 0.01), hearing disability (*P* = 0.01), diabetes mellitus (*P* = 0.02), Independent Living Index (*P* = 0.01), physical performance (*P* = 0.03), body pain (*P* = 0.03), perceived health status (*P* = 0.001), ability to emotional role (*P* = 0.03), Positive Mood Index (*P* = 0.001), Negative Mood Index (*P* = 0.001), Formal Social Cohesion (*P* = 0.001), Informal Social Cohesion (*P* = 0.001), general health (*P* = 0.04), and vitality (*P* = 0.04) gained meaningful correlation with the output class (QoL among the elderly) statistically at *P* < 0.05. Other variables, including gender, marital status, educational level, place of residence, gastrointestinal diseases, nervous diseases, skin diseases, and other diseases with *P* > 0.05, were excluded from the study.

### Binary logistics regression analysis

The results of detecting the hybrid correlational between all variables to predict the QoL among the elderly using the Enter method of the BLR are shown in Table 2.

**Table 2:** The correlation effects of all crucial factors affecting the elderly’s QoL

Variable	B (correlation)	Odd ratio (OR)	95% CI for OR	*P*-value
Age	0.25	0.665	[0.45–0.86]	0.01
Heart diseases and high blood pressure	0.32	1.125	[0.850–1.442]	0.01
Musculoskeletal diseases	0.21	0.43	[0.221–0.0662]	0.11
Diabetes mellitus	0.36	1.452	[0.755–1.223]	0.04
Respiratory diseases	0.27	0.977	[0.853–1.452]	0.04
Cancer	0.22	0.55	[0.324–0.855]	0.03
Mental disorders	0.31	0.64	[0.421–0.993]	0.04
Visual disability	0.26	0.324	[0.228–0.471]	0.09
Hearing disability	0.16	0.541	[0.324–0.667]	0.18
Independent Living index	0.14	0.456	[0.232-.0796]	0.01
Physical performance	0.26	0.335	[0.192–0.441]	0.03
Body pain	0.12	0.171	[0.085–0.193]	0.09
Perceived health status	0.31	0.846	[0.554–1.067]	0.1
Ability to emotional role	0.16	0.443	[0.321–0.554]	0.04
Positive mood index	0.17	0.995	[0.835–1.379]	0.01
Negative mood index	0.11	0.542	[0.342–.0871]	0.02
Formal social cohesion	0.35	0.742	[0.835–1.379]	0.03
Informal social cohesion	0.36	0.778	[0.536–.0831]	0.01
General health	0.13	0.325	[0.245–0531]	0.12
Vitality	0.14	0.227	[0.835–1.379]	0.17

This table shows the BLR enter method containing the prediction power of input variables for the elderly’s QoL. In this table, B shows the correlation between input variables and the output class. The odd ratio and confidence of the odd ratio (CI) indicate the odd of input variables and the odd of input variables with 95% confidence intervals. By considering the *P* < 0.05 as a statistically significant level, the variables of age (*P* = 0.01), heart diseases and high blood pressure (*P* = 0.01), diabetes mellitus (*P* = 0.04), respiratory diseases (*P* = 0.04), cancer (*P* = 0.03), mental disorders (0.04), Independent Living Index (*P* = 0.01), physical performance (*P* = 0.01), ability to emotional role (*P* = 0.04), Positive Mood Index (*P* = 0.01), Negative Mood Index (*P* = 0.02), Formal Social Cohesion (*P* = 0.03), and Informal Social Cohesion (*P* = 0.01) were as the factors affecting the elderly’s QoL in the presence of other independent factors. Other variables with a correlation at *P* > 0.05 were excluded in this step. Although they have a significant, simple correlation with QoL, by excluding them and using the variables with significant correlation in the presence of other variables, the probability of algorithm overfitting would be decreased to a minimal level in a high-performance state.

### Models assessment

**Fig. 2:**
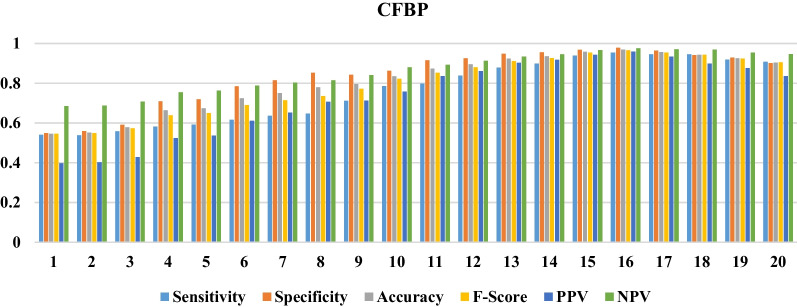
The performance of the CF-BP in various numbers of neurons in the hidden layer

**Fig. 3:**
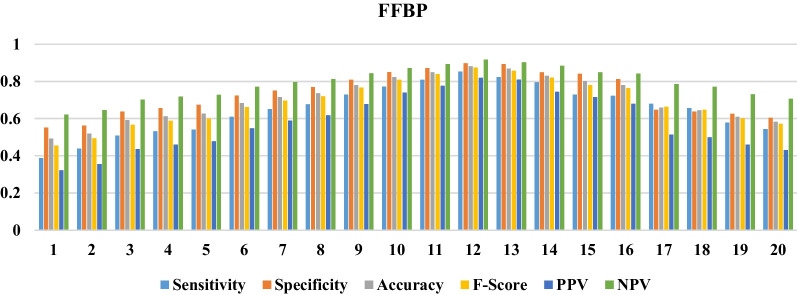
The performance of the FF-BP in various numbers of neurons in the hidden layer

**Fig. 4:**
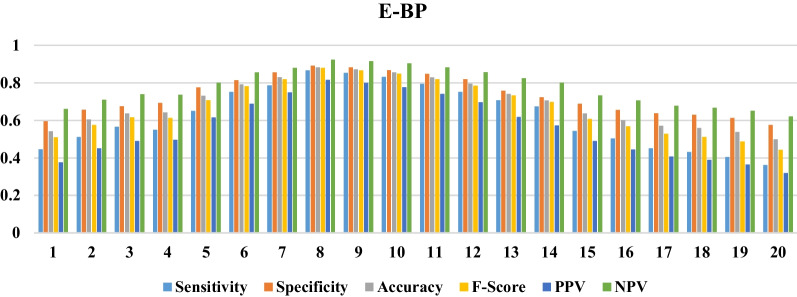
The performance of the E-BP in various numbers of neurons in the hidden layer

The results of comparing various modes of ANN by increasing the number of neurons to 20 in hidden layers are represented in Figs. 2, 3 and 4.

**Fig. 5:**
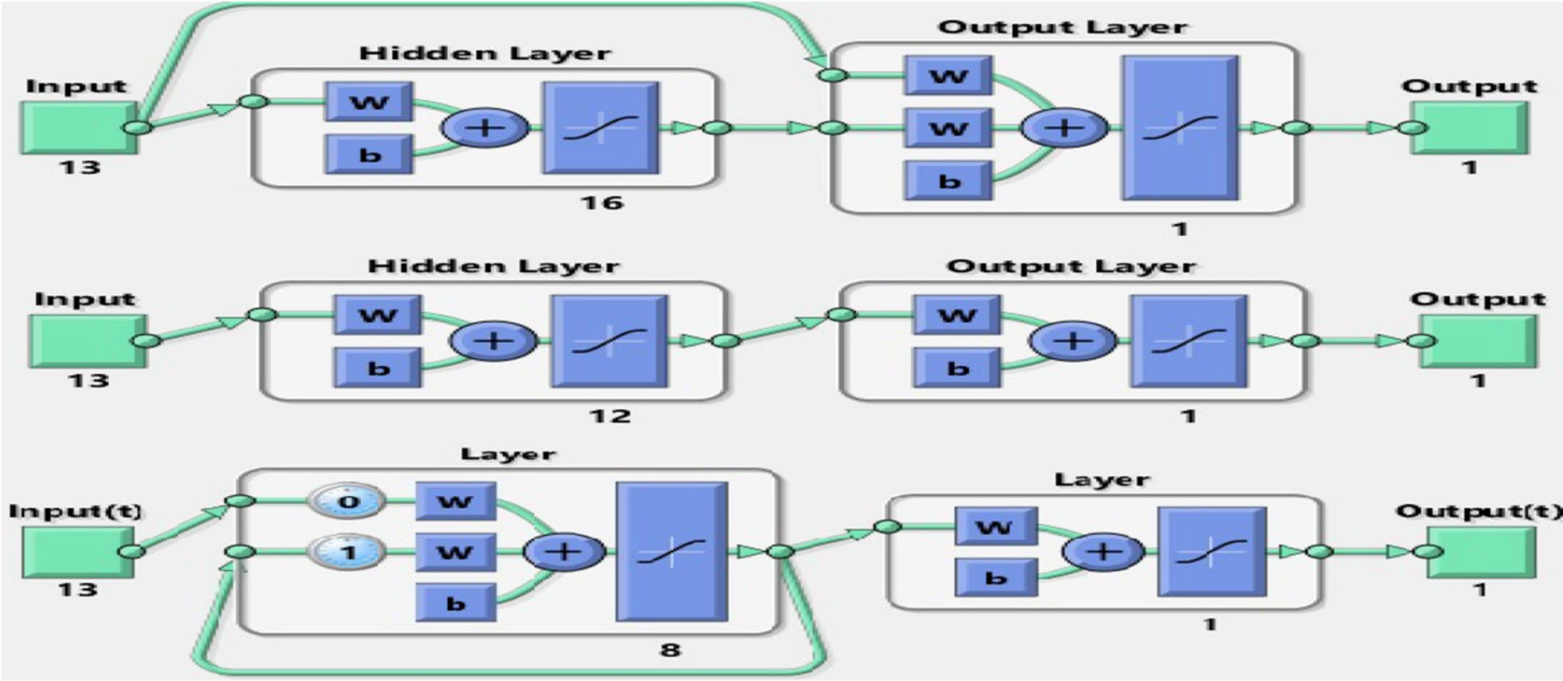
The selected configuration of each ANN algorithm

Based on information obtained from Figs. 2, 3 and 4, the CF-BP with the configuration of 13-16-1 with sensitivity = 0.95, specificity = 0.97, accuracy = 0.96, F-Score = 0.96, PPV = 0.95, and NPV = 0.97 gained a better performance than the other configurations having different neurons in the hidden layer. The FF-BP with sensitivity = 0.85, specificity = 0.89, accuracy = 0.88, F-Score = 0.87, PPV = 0.81, and NPV = 0.92 in the structure of 13-12-1 obtained better performance than the other modes pertained to this configuration type. Also, the EBP in the configuration of 13-8-1 with sensitivity=0.86, specificity = 0.89, accuracy = 0.88, F-Score = 0.87, PPV = 0.81, and NPV = 0.92 obtained the best performance for predicting QoL among the elderly. The structures with the best performance belonging to each ANN are shown in Fig. 5.

Generally, the CF-BP with sensitivity = 0.95, specificity = 0.97, accuracy = 0.96, F-Score = 0.96, PPV = 0.95, and NPV= 0.97 in the configuration of 13-16-1 had better performance than other models for predicting the QoL among the elderly. We compared three selected configuration models using each algorithm’s ROC curve and error rates to investigate the overfitting states in train, test, and validation modes.

### Overfitting investigation of selected ANN configuration

**Fig. 6:**
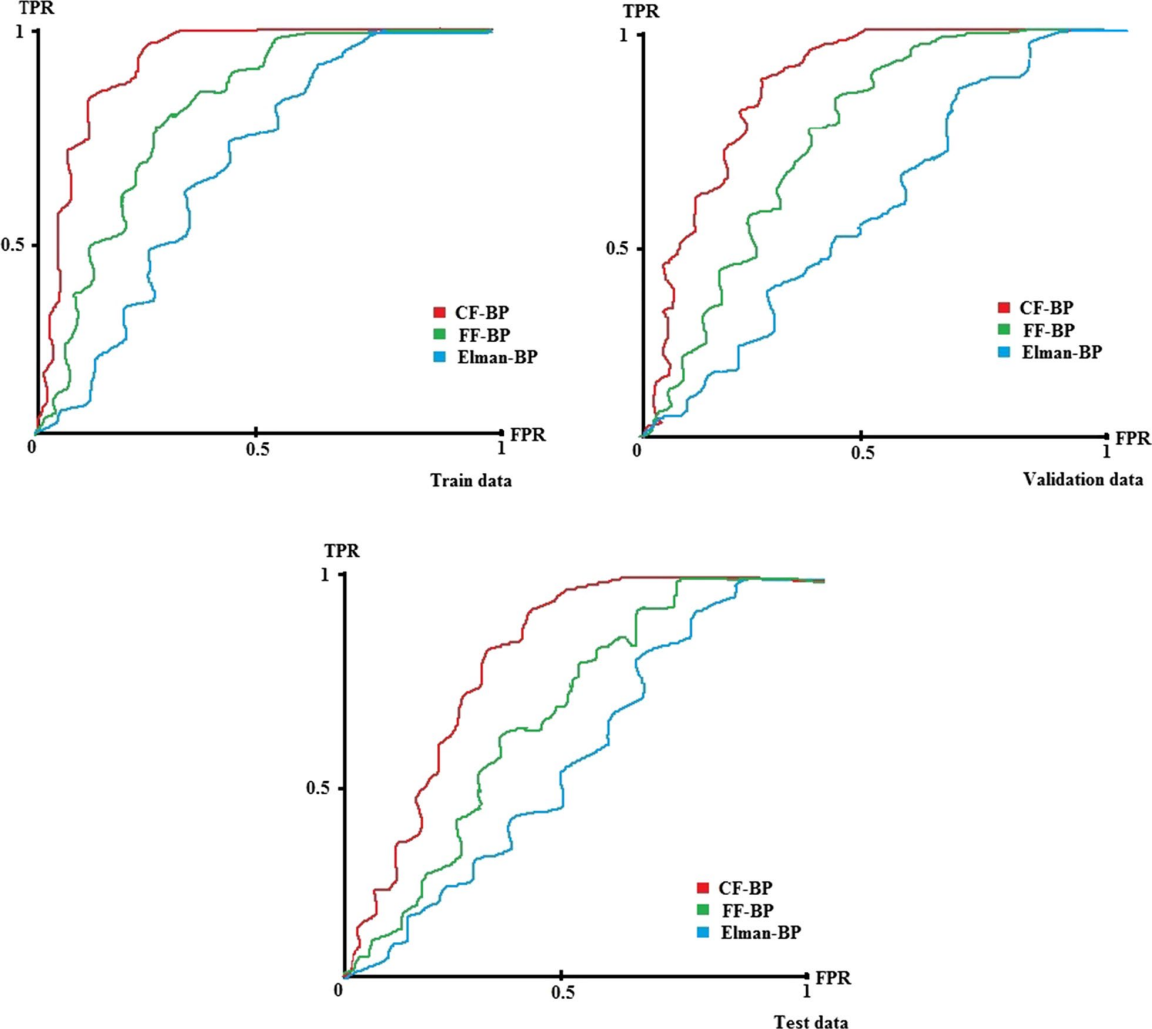
The performance of selected ANN configurations in all modes

The results of comparing the ROC curve of the best configurations in each train, test, and validation mode are shown in Fig. 6 (the vertical vertices show a True Positive Rate, and the horizontal vertices show a False Positive Rate).

**Fig. 7:**
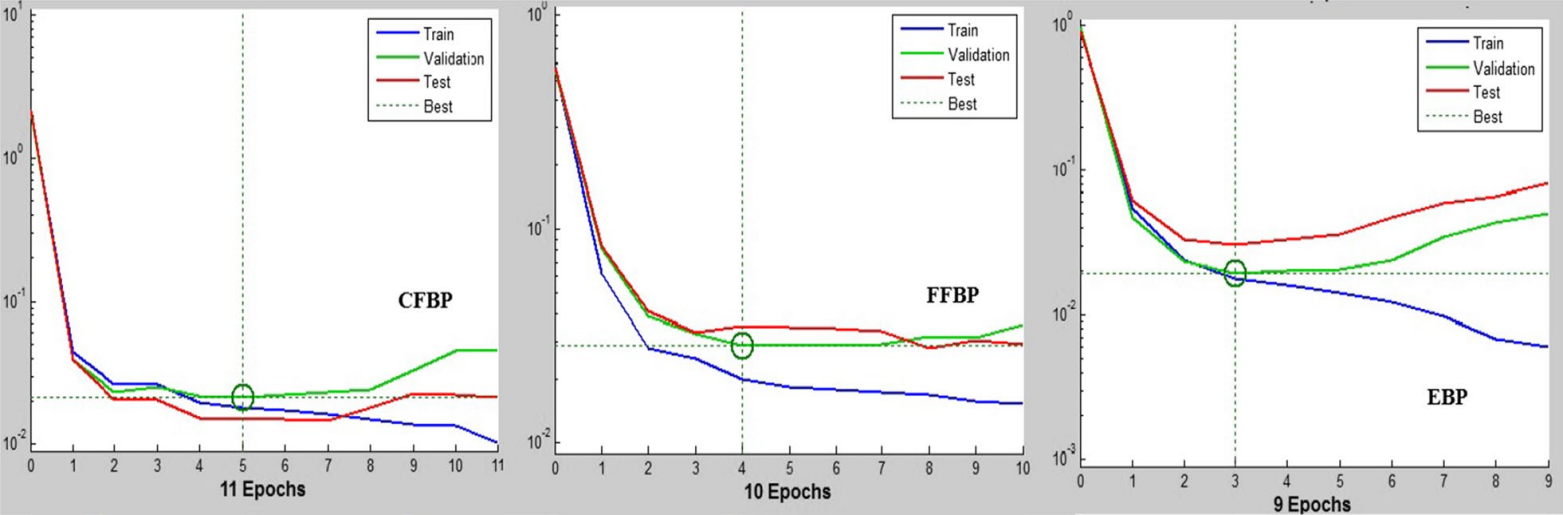
The binary cross entropy loss function of selected ANN configurations

Based on Fig. 6, the CFBP with AUC-train=0.913, AUC-validation=0.906, and AUC-test = 0.855 was the best model for predicting the QoL among the elderly in the train, validation, and test modes. The E-BP with AUC-train = 0.713, AUC-validation = 0.705, and AUC-test = 0.602 gained the lowest performance for predicting QoL among the elderly. Also, the AUC rate in the test mode was decreased more than in other selected ANN configurations. The error rates of all selected configurations of ANNs during training time are shown in Fig. 7.

**Fig. 8:**
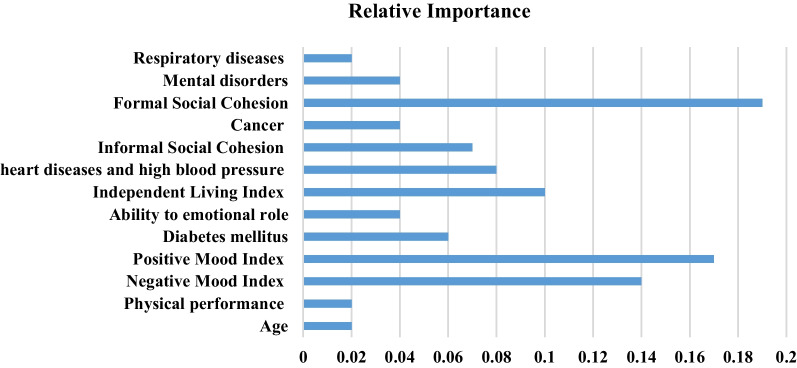
The relative importance of the elderly’s QoL predictors by CFBP

Based on Fig. 7, the E-BP with binary cross entropy loss function_-validation_ = 0.02 during the 9th iteration of the ANN training had a slight error for predicting the QoL among the elderly. Still, the difference between the train validation and test modes is more significant than in other models. (The error of train and validation modes are closed to 10^−2^, but the test is closed to 10^−1^). So the overfitting probability in this algorithm is more than in others with this difference. On the contrary, the CFBP with binary cross entropy loss function in all train, validation, and test modes closed to 10^-2^ gained minor errors in the test state for predicting QoL among the elderly. The predictors are listed based on their relative importance, recognized by CFBP in Fig. 8.

### Factors assessment for elderly’s QoL prediction by ANN and CDSS designed

**Fig. 9:**
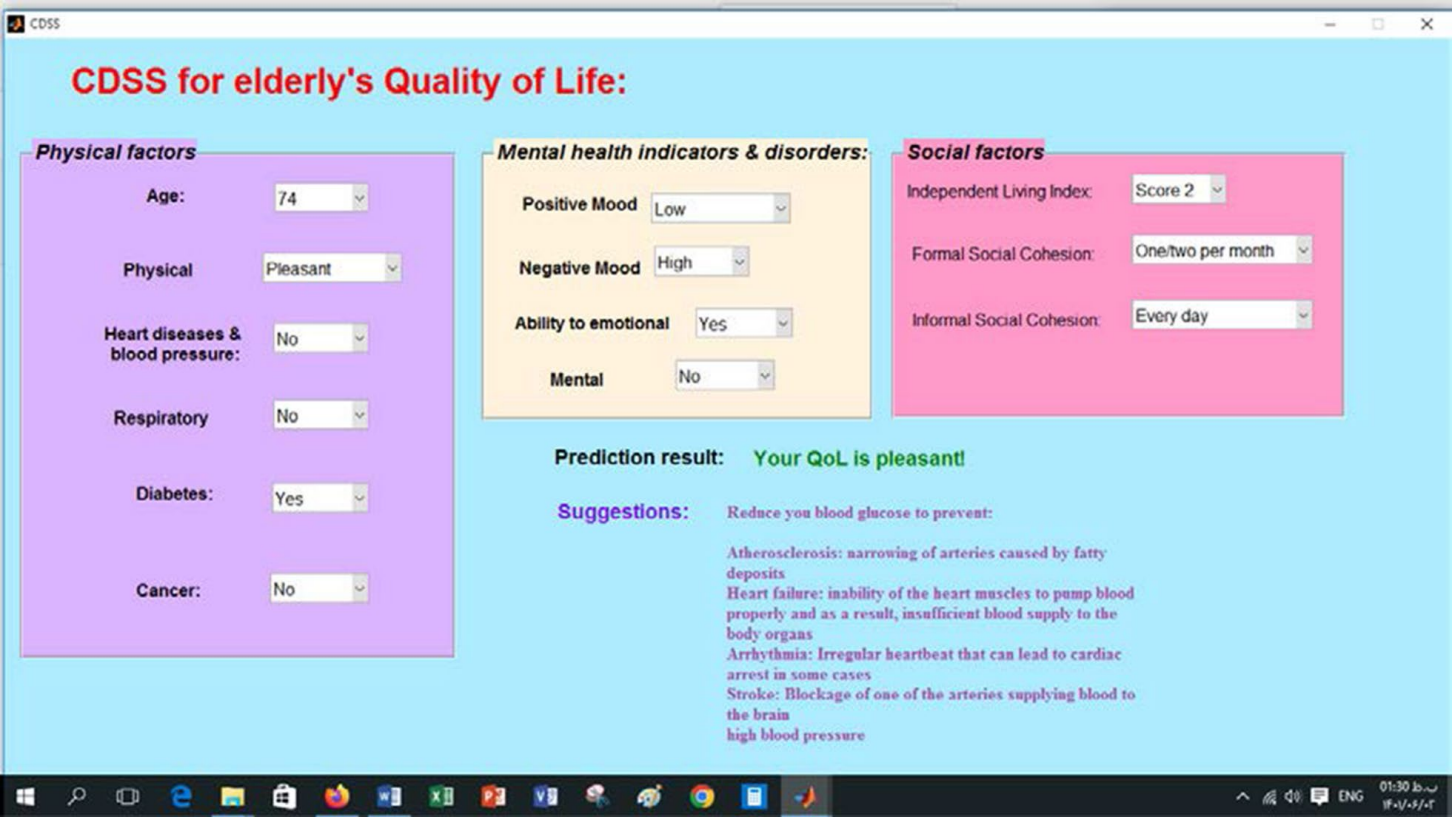
A schema of CDSS designed for the elderly’s QoL prediction

Based on Fig. 8, Formal Social Cohesion with relative importance = 0.19, Positive Mood Index with relative importance = 0.17, and Negative Mood Index with relative importance = 0.14 were considered the best factors affecting the QoL among the elderly by CFBP. The predictors of respiratory diseases, age, and physical performance with relative importance = 0.02 were the least important in this respect. The CDSS (Fig. 9) for predicting QoL among the elderly using essential predictors was developed in MATLAB R2013-a environment. The people can enter the factors associated with QoL among the elderly, and the system predicts the elderly’s QoL.

### External validation cohort

The results of testing our prediction system using the confusion matrix are shown in Table 3.

**Table 3:** The confusion matrix to determine the elderly's QoL

	Predicted QoL	Predicted non-QoL
Real QoL	52	13
Real non-QoL	12	70

**Fig. 10:**
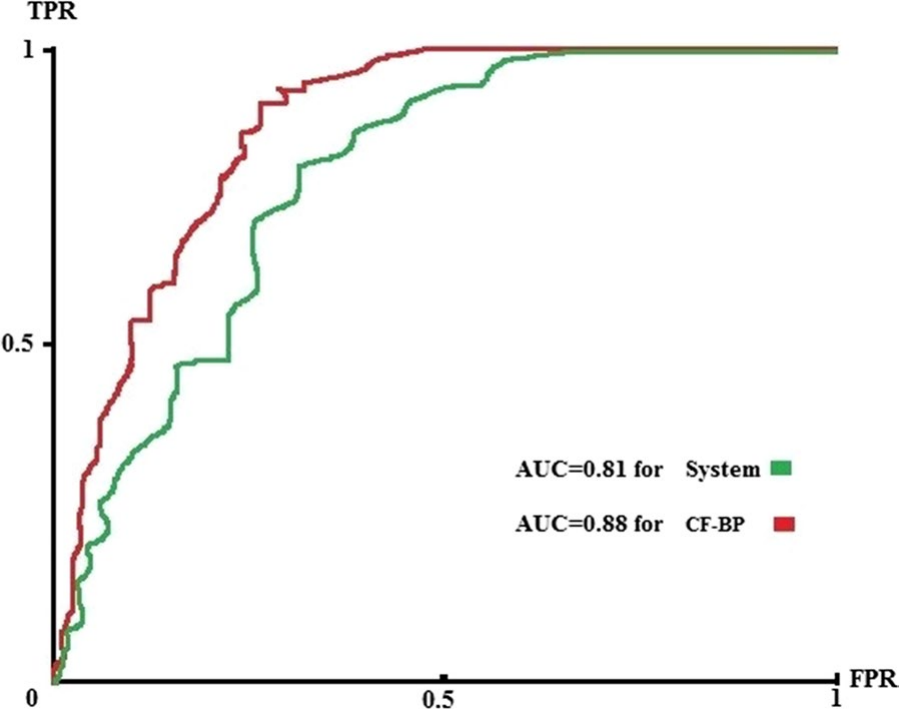
Comparing the AUC of the system and CF-BP

In this table, real QoL and real non-QoL are the real cases gathered for the external validation cohort. Predicted QoL and predicted non-QoL are the cases predicted by the system. Among 65 elderly’s QoL, 52 cases were correctly classified by the system, and 70 cases from 82 real cases pertained to elderly's non-QoL were correctly classified. Based on confusion matrix and Eqs. 1–6, the prediction system with sensitivity = 0.8, specificity = 0.85, accuracy = 0.83, F-Score = 0.825, PPV = 0.812, and NPV = 0.84 obtained pleasant performance in predicting the elderly’s QoL. The results of comparing the performance of CF-BP with the structure of 13-16-1 and the prediction system using the ROC curve are depicted in Fig. 10.

Based on Fig. 10, our prediction system with the AUC of 0.81 compared to the CF-BP with the AUC=0.88 did not get much-reduced performance in predicting the elderly’s QoL. This performance for prediction shows the relatively favorable generalizability of the prediction system for the elderly’s QoL in these two centers.

## Discussion

The QoL is a complicated concept that pertains to the physical, psychiatric, and social aspects of the health status of people. Attention to these aspects and creating a solution for enhancing them in elderly life has a significant role in increasing the QoL. So this study aimed to develop a CDSS based on the ANNs algorithm as an ML approach to forecast the QoL among the elderly. In this study, we selected the highly correlated variables for predicting the elderly’s QoL using the BLR with the Enter method. This way, all variables were entered into the model in one step. This method allowed us to choose variables with high correlation in the combination of other variables and the performance of the model reached its highest level when using them. To build the predictive model for predicting the elderly’s QoL, we used the three BP types of the ANN, including the CF-BP, FF-BP, and E-BP algorithms. The best configuration in each algorithm was obtained using sensitivity, specificity, and accuracy. Then, we assessed the selected ANN configurations in each train, test, and validation mode using the ROC curve and binary cross entropy loss function. Finally, the best predictive for QoL among the elderly was obtained, and the CDSS was designed in MATLAB V 2013-a environment. So far, little research has been conducted in the elderly's QoL prediction field that has investigated all aspects of physical, psychiatric, and social factors, especially in the ML domain; in other words, they were studied separately.

Oliveira et al. investigated the automatic classification of disabilities among the elderly using the ADL variable. In that study, multiple correspondence analysis, k-means algorithm, association rules, and network analysis were used for classification, and they resulted that 6.2 of the elderly have a discriminant disability [45]. In the current study, we analyzed the physical, psychiatric, and social factors using the BLR algorithm and investigated the effects of these variables on the elderly’s QoL. Lee et al. studied the biomarkers associated with the QoL among the elderly using ML algorithms. Multivariate regression was used to identify the importance of determining factors for predicting the QoL among the elderly and the relationship of these factors with physical and biological markers. The results showed that the variable of handshaking strength, with a beta correlation of 1.71 for men and 1.74 for women, is the essential factor concerning physical indicators and the variables of handshaking strength and walking speed. The number of chronic diseases was considered the most important biological factor related to mental indicators predicting the quality of life of the elderly [46]. In the current study, the physical and mental factors are crucial for predicting QoL among the elderly. Na et al. conducted a study to predict the 2-year future of the cognition of the elderly community using ML techniques. The Gradient Boosting algorithm was used to build the prediction model and data analysis. The results showed that the variable of age is the crucial factor affecting cognitive prediction. The algorithm gained sensitivity = 96.7%, specificity = 82.5%, and AUC = 0.92 to predict the cognitive trends [47].

In this study, the BLR considered the age at *P* = 0.01 as the critical factor affecting the QoL among the elderly. Byeon et al. developed a model for predicting the social participation of the elderly using ANN and Quest algorithms. The performance evaluation showed that the ANN gained the MSE = 240.11 and the AUC = 0.718. Also, the accuracy of the training, testing, and validation data set was 75.8%, 72.8%, and 75%, respectively. The performance evaluation of the QUEST decision tree showed an accuracy of 75.4%, which was obtained by ten cross-validations. The age with RI = 0.106 and subjective health status were essential factors affecting social participation [48]. In this study, the age variable was considered the important factor for predicting the QoL among the elderly.

In this study, we used physical and psychological disabilities, disorders, and social factors to predict the elderly QoL, contrary to most other studies that usually investigate physical and/or psychological factors. The use of all dimensions of factors affecting the elderly’s QoL increases the performance and generalizability of prediction models that are considered in this study.

In the current study, we developed the ANN-based CDSS as an ML technique to build the prediction system for QoL among the elderly based on physical, mental, and social factors. This system can predict the elderly’s QoL by assessing elderlies using these factors and recommend the best solution to modify these factors and increase the elderly’s QoL. Therefore, this system can provide the best clinical evidence-based solutions for gerontologists to increase the QoL in the latest years of age in the elderly. Also, we used the simple correlation and BLR as the FS process to get the most relevant factors affecting the elderly’s QoL. This method caused minimal overfitting the ANN algorithms after we tested them using ROC and binary cross-loss function. The limitations of our study are the sophistication of the definitions of QoL. By introducing this concept, we clarified it by defining the quality of life with different dimensions. Because of the incompleteness of some records with missing data-pertained features, we were forced to replace them through predicted values by the KNN algorithm. This process may affect the generalizability of the system using artificial data. Some variables were qualitative in the database, which might reduce the algorithm's performance. In this study, we used more dimensions of factors for QoL among the elderly than in other studies. However, using more variables from the present study in each dimension of physical, psychological and social factors can increase the generalizability of the model. Due to the extent of the QoL concept, more variables may be existed for predicting QoL among the elderly. Their use increases the performance of different ML algorithms compared to our study. For the future, we propose to examine the real data by extracting all data from the records, collecting more data from more elderly centers to increase the generalizability of the system, and using more quantitative data and more features affecting the elderly's QoL for higher prediction precision. However, the impact rate of this intelligent system in increasing the QoL and improving modifiable factors affecting QoL among the elderly is one question that can be investigated in future studies with interventional methods.

## Conclusion

Today, using a solution to predict the QoL can promote life satisfaction and lifestyle among the elderly. The study shows that analyzing the predictive factors affecting the elderly's QoL as FS process and selecting the most relevant features for the prediction can increase the ML algorithms’ performance and prevent the models from overfitting. The social, mental, and physical factors have a potential role in building the prediction model for the elderly’s QoL with high performance with generalizability. Based on the study's results, the ML and, specifically, the ANN algorithms can be used as pleasant prediction models for QoL among the elderly. The CDSS based on the ANNs can assist gerontologists in predicting the QoL among the elderly by providing their clinical and evidence-based judgment. They can present the best solutions to the elderly to modify their lifestyle through disabilities and disorders based on the results of the predictive system. This subject decreases dependence on others and increases confidence and QoL among elderlies in the latest years of their life.

## Data Availability

The datasets generated and/or analyzed during the current study are not publicly available to the privacy concerns of research committee but are available from the corresponding author on reasonable request.

## Abbreviations

QoL: Quality of life
CDSS: Clinical decision support system
ANN: Artificial neural network
ML: Machine learning
BLR: Binary logistics regression
B-P: Back-propagation
CF-BP: Cascade forward back-propagation
FF-BP: Feed forward back-propagation
E-BP: Elman back-propagation
PPV: Positive predictive value
NPV: Negative predictive value
